# Communication and information needs of patients with cancer in Ghana: A scoping review

**DOI:** 10.1371/journal.pone.0343094

**Published:** 2026-02-12

**Authors:** Kofi Gyasi Agyei, Ahmed-Rufai Yahaya, Natalie LeBlanc, Kah Poh Loh, Sally A. Norton

**Affiliations:** 1 School of Nursing, University of Rochester, Rochester, New York, United States of America; 2 Penn State Ross and Carol Nesse College of Nursing, University Park, Pennsylvania, United States of America; 3 Division of Hematology and Oncology, Department of Medicine, Wilmot Cancer Institute, University of Rochester Medical Center, Rochester, New York, United States of America; University Hospital Cologne: Uniklinik Koln, GERMANY

## Abstract

**Background:**

Patients with serious illnesses, especially those with cancer, require information and good communication throughout their care. Effective communication is pivotal for quality cancer care, leading to improved quality of life, reduced anxiety, enhanced patient satisfaction, and adherence with treatment recommendations. Considering this, programs including serious illness conversation guides have been developed and implemented internationally (e.g., in the US) to improve communication skills in oncology care. Although many low- and middle-income countries are experiencing a growing cancer burden in both incidence and mortality, prognosis and treatment options are rarely discussed, which may be due to a lack of culturally sensitive guidelines in the African context. Notably, Ghana is facing a concerning increase in cancer prevalence and incidence. However, there is a paucity of information about the communication and information needs of patients with cancer in Ghana. This review will provide critical insights to guide the development or adaptation of culturally sensitive guidelines/frameworks appropriate for the Ghanaian context, with potential applicability to other African countries facing similar sociocultural and health system dynamics.

**Objective:**

This scoping review sought to explore and synthesize existing evidence on the communication and information needs of patients with cancer in Ghana.

**Methods:**

A systematic search was undertaken across PubMed, Web of Science, CINAHL, EMBASE, and PsycINFO databases using keywords and MeSH terms related to cancer, communication/information, and Ghana, employing Boolean operators “OR,” “AND.” The scoping review was guided by the five-stage methodological framework proposed by Arksey and O’Malley. The Preferred Reporting Items for Systematic reviews and Meta-Analyses extension for Scoping Reviews (PRISMA-ScR) was further adopted to ensure comprehensive reporting.

**Results:**

Eight studies published between 2019 and 2023 met the inclusion criteria. Two major themes with sub-themes were identified including (1) information and communication experiences ((i) concealed information, (ii) disruption in communication, (iii) breaking bad news, (iv) power asymmetry-deference to healthcare providers), and (2) communication and information needs and expectations ((i) information on disease conditions, (ii) information on cancer treatment, (iii) information on prognosis, (iv) ongoing information, (v) involvement in decision-making).

**Conclusions:**

Ghanaian patients with cancer strongly desire information regarding their condition and expect to be involved in decision-making. Despite patients with cancer’s desire for comprehensive information and shared decision-making, gaps in healthcare communication were evident, including limited accessibility to information, inadequate explanations by healthcare providers, and a lack of patient involvement in decision-making. Addressing these gaps requires tailored interventions to improve healthcare provider-patient communication.

## Introduction

Individuals with serious illnesses require adequate information and effective communication throughout their disease trajectory [1]. Effective communication is essential for high-quality end-of-life care, particularly for individuals diagnosed with serious illnesses like cancer, due to the health risks and substantial burden it places on caregivers. Addressing patient health literacy needs, prognosis, and patients’ values or goals of care is integral for person-centered care, especially in serious illness [2]. It is also essential for individuals with cancer as it enables them to receive timely and pertinent information, particularly about their prognosis and treatment options, especially during the end-of-life stage, which ensures informed decision-making. It can help reduce resource utilization, alleviate patient stress and anxiety, promote more goal-concordant care, and enhance clinicians’ experiences in providing care [3,4].

Despite the importance and the desire of patients and their families to engage in communication with healthcare providers, a significant portion of patients with advanced cancer, estimated at about half, are unaware of their prognosis as they approach the end of life [5]. Moreover, only about one-third of patients with serious illness report engaging in goals of care conversations with their healthcare providers [6]. These conversations, when they occur, are mostly during the end-of-life phase, and some key aspects, including emotional, cultural, and psychosocial needs, are not addressed [7]. This underscores a gap in effective communication between healthcare providers and patients. Considering this, programs have been developed and implemented regarding communication skills in oncology care [8,9]. To an extent, these programs have been declared mandatory for clinicians specializing in cancer care [8]. In the United States, a serious illness conversation guide was developed by Ariadne Labs to guide conversations in oncology care [4]; however, such guidelines are lacking in some low-and middle-income countries, including Africa. Other guidelines developed for having difficult conversations with patients include the Airway, Breathing, Circulation, Disability, Exposure (ABCDE) [10], and the SPIKES protocols [11].

Although many low- and middle-income countries, especially those in Africa, are experiencing an increase in cancer burden, discussions about death and dying, prognosis, and treatment options are rarely undertaken [12]. Notably, Ghana, located in West Africa, is facing a concerning increase in cancer incidence, prevalence, and mortality rates, making it an emerging public health issue in the country [13]. Approximately half of the patients seeking care at major cancer centers in Ghana are reported to present with advanced or late-stage (stage III and IV) cancers [14,15]. This may be due to the limited resources to perform cancer screening and hence make a timely diagnosis, challenges in the patient referral process, medical pluralism, and healthcare costs. These resources are also exclusively available in the largest southernmost cities, the country’s capital (Accra), and Kumasi in the southern central part of Ghana [16].

Notwithstanding the rise in cancer cases in Ghana, little is known about the communication and information experiences and needs of patients with cancer in Ghana. Considering the importance of effective communication in cancer management, gaining a better understanding of the communication and information experiences, needs, and preferences of patients with cancer in the Ghanaian context is necessary. This could facilitate the development of services tailored to the local needs, inform efficient resource allocation, and guide effective communication interventions to meet the needs of patients with cancer and clinicians in Ghana. Considerations of cultural context by which health and illnesses communication takes place is important because studies have indicated the influence of sociocultural context on communication needs and preferences [17,18]. Moreover, a recent scoping review conducted by DeBoer et al. [17] assessing clinical communication in cancer care in Africa suggested that patient-centered communication in Africa should be explored. This scoping review was therefore conducted to identify and synthesize existing evidence on the communication and information needs of patients with cancer in Ghana. Specifically, the scoping review sought to answer the question: What are the communication and information experiences and needs of patients with cancer in Ghana? The findings of this review can inform a culturally tailored communication guide or framework for settings like Ghana.

## Methods

Scoping reviews are structured methodologies used to examine broad research areas by incorporating studies with various designs to map existing literature and identify the range and nature of available evidence [19]. This scoping review was conducted to explore the existing literature on information needs and communication in cancer care in Ghana. This scoping review was conducted following the five-stage methodological framework proposed by Arksey and O’Malley [19], expanded upon by Levac et al. [20]. The Preferred Reporting Items for Systematic reviews and Meta-Analyses extension for Scoping Reviews (PRISMA-ScR) checklist (S1 Appendix) was further adopted to ensure comprehensive reporting [21]. The five stages in the framework include: identifying the research question, identifying relevant studies, charting the data, and summarizing, synthesizing, and reporting results. There is no published protocol for this scoping review.

### Identifying the research question

This scoping review sought to answer the following question:

1. What are the communication and information experiences and needs of patients diagnosed with cancer in Ghana?

### Identifying relevant studies

A preliminary systematic literature search was undertaken between September 2024 and January 2025 across the CINAHL, PsycINFO, PubMed, EMBASE, and Web of Science databases based on recommendations and assistance from a librarian at the School of Nursing, University of Rochester. This helped refine the search terms and conclude on the most suitable strategy. The final comprehensive literature search across the databases was conducted in May 2025. The keywords and Medical Subject Headings (MeSH) terms used in the databases were aligned with the population, concept, and context framework presented in **Table 1**. Boolean terms or/and were used to enhance the comprehensiveness and specificity of the search strategy with the assistance of a librarian [22]. The search strategies used for all the databases are presented in S2 Appendix.

**Table 1:** Conceptual statements and corresponding keywords/MeSH terms mapped onto the PCC framework.

Population	Concept	Context
Patients with cancer	Communication/communication experiences Communication and information needs	Ghana
Cancer (“neoplasm*”, “cancer*”, “oncology”, “cancer treatment”, “malignancy”, “tumor”, “carcinoma”, “sarcoma”)	Communication (“health communication”, “communication”, “nonverbal communication”, “verbal communication”, “written communication”, “oral communication”) Information (“information literacy”, “consumer health information”, “health information”, “information seeking”, “information”, “information needs”)	Ghana

Table 1 presents the keywords and MeSH terms used for the review.

### Selecting the studies

**Fig 1:**
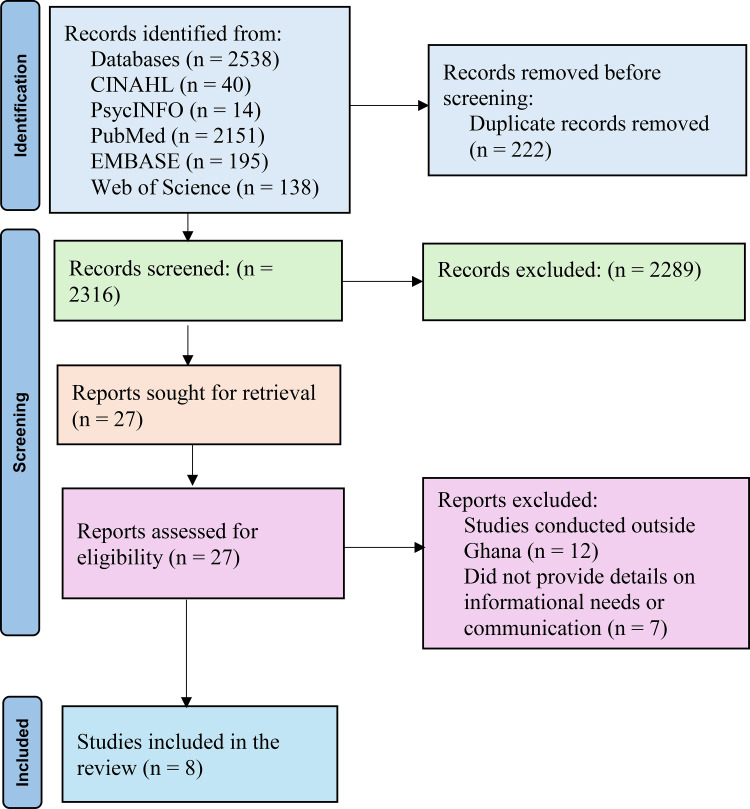
PRISMA flow chart showing the study selection process. A total of 2,538 records were identified, with eight studies meeting the final inclusion criteria after full-text screening.

Two thousand five hundred and thirty-eight (2538) studies were identified from the databases through the search using the search strategy developed for the scoping review. Two hundred and twenty-two (222) duplicates were removed using the EndNote reference manager. Two thousand two hundred and eighty-nine (2289) articles were removed through title and abstract screening. Full-text articles of the remaining studies [27] were assessed for eligibility, and eight met the inclusion criteria. The first (KGA) and another author (ARY) undertook the screening and selection process. They independently reviewed the articles and then engaged in peer debriefing to discuss dissenting decisions, reaching a consensus regarding the included studies. The inclusion and exclusion criteria used for the review are presented in Table 2. The search results summary is presented in **Fig 1** using the PRISMA flow chart.

**Table 2:** Inclusion and exclusion criteria.

Inclusion Criteria	Exclusion Criteria
• Peer-reviewed original research • English Language only • Studies that adopted either quantitative, qualitative, or mixed methods approach • Studies that reported information needs of patients with cancers (any stage), or communication practices among healthcare providers and patients with cancers (both patients and healthcare providers perspectives) • Studies conducted in Ghana • Studies published from 2012 to 2025	• Review papers • Studies that did not provide complete data • Opinion papers, conference proceedings, theses, and dissertations.

Table 2 presents the inclusion and exclusion criteria used for screening the studies.

### Charting the data

Two authors (KGA and ARY) extracted data from the included studies using a standardized extraction form (Excel sheet) developed specifically for the review. The data extracted from the studies included the author(s), year of publication, study aim(s), study design, sample, and key findings such as the experiences and information needs of patients with cancer (Table 3).

**Table 3:** Characteristics of included studies (N = 8).

Author(s)/Year	Study Design	Sample/Setting	Study Aim(s)	Key Findings
Agyemang et al. (2021) [26]	Qualitative Ethnographic approach Observations and Semi-structured interviews	N = 31 Women diagnosed with breast cancer (Curative intent to stage III), patients-nominated family members, doctors, and nurses. Breast clinic in a public teaching hospital	To explore the socio-cultural factors influencing treatment decision-making for women with breast cancer in Ghana.	Due to language barriers and unequal patient-provider relationships, essential information (disease stages and treatment) is hidden from patients. Absence of appropriate information materials.
Akuoko et al. (2022) [24]	Quantitative Cross-sectional study design. Self and researcher administered surveys	N = 176 Women diagnosed with advanced breast cancer (Stage III and IV). One public hospital and a private institution involved in the management of breast cancer.	To assess the needs (perceived and expressed) of women with advanced breast cancer.	The most reported supportive care needs were related to information needs (96.6%). The information needs included: being informed about benefits and side-effects of treatments before choosing a treatment plan (90.3%), being informed about what to do to get well (94.9%), having a healthcare provider to talk about the condition and management (92.1%).
Akuoko et al. (2022) [23]	Qualitative Qualitative descriptive design Semi-structured interviews.	N = 13 Healthcare professionals and key informants (disciplines- medicine, nursing, counseling, dietitian, physician assistant, social worker) who support women with advanced breast cancer. Oncology directorate of a teaching hospital, a private hospital, and two NGOs.	To explore the perspectives of healthcare professionals and key informants concerning the supportive care needs of women with advanced breast cancer.	Healthcare professionals anticipate that women with advanced breast cancer require information about their disease process (knowledge, risk factors/causes of cancer) and services/treatment process (treatment options, prognosis/outcomes, and side effects/complications of the disease).
Boadi et al. (2021) [29]	Quantitative Cross-sectional design. Survey	N = 75 Female patients diagnosed with breast cancer. Breast cancer unit of two health facilities in the capital city of Ghana.	To investigate the information needs of women living with breast cancer in Ghana.	Patients prioritized diagnostic information as their greatest need, followed by information on physical care, treatment information, psychosocial information, and disease-specific information. There was less emphasis on preventive information.
Hobenu and Naab (2022)	Qualitative Exploratory descriptive study design Semi-structured interviews	N = 15 Women with advanced cervical cancer. Radiotherapy center in a teaching hospital	To explore the experiences of women diagnosed with advanced cervical cancer as they access a specialist healthcare.	The study revealed information gaps as most participants indicated that they were not informed about the side effects of treatment they received and were also given partial information about condition when they asked their healthcare providers about their disease. Participants perceived a lack of knowledge from healthcare staff.
Kudjawu and Agyeman-Yeboah (2021)	Qualitative Exploratory descriptive design Semi-structured interviews	N = 8 Women diagnosed with breast cancer. Oncology directorate of a teaching hospital.	To explore the experiences of women with breast cancer.	The study highlighted that participants’ major challenges were limited access to information from healthcare providers, insufficient resources, and financial constraints.
Kugbey et al. (2019)	Quantitative Cross-sectional design Survey	N = 205 Women living with breast cancer. Oncology directorate of a teaching hospital.	To assess the direct and indirect influences of access to health information and health literacy on the quality of life of women with breast cancer.	Some participants (2.4% and 7.8%) were very dissatisfied and dissatisfied, respectively, with the amount of information received from healthcare providers. Some participants also indicated that their concerns were somehow (17.6%), and not at all (3.4%), addressed.
Mensah et al. (2023)	Qualitative Exploratory descriptive design Semi-structured interviews	N = 15 Individuals with histology-confirmed cancer diagnoses receiving treatment for a minimum of 6 months. Oncology directorate of a teaching hospital.	To explore patients with cancer’s expectations of palliative care services and assess the barriers that hinder the utilization of palliative care.	Patients with cancer expect clear and meaningful communication regarding their condition and prognosis. They expect active participation in decision-making.

### Summarizing, synthesizing, and reporting results

The scoping review incorporated both numerical summary data and thematic analysis [20,23]. A thematic analysis of the key findings extracted from the included studies was undertaken. The charted data were systematically examined using an inductive approach to identify recurring patterns and salient themes that reflected the central findings across the studies [23,24].

## Results

### Study characteristics

Eight articles met the inclusion criteria of the scoping review. Four of the included studies involved patients with advanced cancers [15,25–27]. Three studies [15,26,28] provided the stages of the cancer of patients ranging from curative intent to stage III [28] and stage III and IV [15,26]. Most of the studies (n = 6) were conducted in teaching hospitals [15,25,27–30]. Most of the studies (n = 5) adopted a qualitative approach [15,25,27–29], while the remaining studies (n = 3) employed a quantitative approach [26,30,31]. All the quantitative studies [26,30,31] adopted a cross-sectional approach using surveys. All the qualitative studies [15,25,27,29] except that of Agyemang et al. [28] adopted a qualitative descriptive design. Agyemang et al. [28] adopted an ethnographic approach. All the qualitative studies [15,25,27–29] employed semi-structured interview as a means for data collection. One study [28] adopted observations in addition to the semi-structured interview. The majority of studies (n = 6) were conducted with patients who had breast cancer [25–31]. One study included patients with varied cancer diagnoses, including advanced breast cancer, metastatic follicular carcinoma, colorectal cancer, cervical cancer, prostate cancer, nasal and sinus cancer, colon cancer, head and neck cancer [15]. Almost all the studies (n = 6) assessed the information needs and experiences from the patients’ perspectives [15,26,27,29–31]. One study assessed the information needs of patients with cancer from the perspectives of healthcare providers [25]. It is important to note that although this study was undertaken on healthcare providers, it assessed what healthcare providers believed were the information needs of patients with cancer and not necessarily how they could communicate with patients nor provide them with the relevant information. Another study also assessed the information needs and experiences from the perspectives of healthcare providers, patients, and their families [28]. Further details of the studies, including the authors, year of publication, study designs, sample, settings, aims, and key findings of the included studies, are presented in **Table 3**.

Table 3 presents the authors, year of publication, study designs, sample, settings, aims, and key findings of the included studies

### Findings

Two main themes – ‘information and communication experiences,’ and ‘communication and information needs and expectations’ – and sub-themes were identified in the included studies **(**Table 4).

**Table 4:** Themes and sub-themes.

Themes	Sub-themes
Information and Communication Experiences	1. Concealed Information 2. Disruption in communication 3. Breaking Bad News 4. Power Asymmetry- Deference to Healthcare Providers
Communication and Information needs and expectations	1. Information on Disease Conditions 2. Information on Cancer Treatment 3. Information on Prognosis 4. Ongoing Information 5. Involvement in Decision-Making

Table 4 illustrates the themes and sub-themes identified

***Theme 1: Information and Communication Experiences***

***Sub-theme 1: Concealed Information***

Some studies revealed that essential information regarding the disease stage, prognosis (expected outcomes), and treatment (options, risks, and benefits) was rarely delivered during patient-clinician interactions [15,27–29].

*“They told me about the hair loss but did not tell me about the general bodily weakness. They also did not tell me about the other side effects…so I thought I was going to die. Due to* *the lack of information, I have always been worried and thinking about what will happen next when I come for treatment”* [27].

It was further indicated that patients’ relatives are sometimes left in the dark. They are not provided with information to help them care for their family member at home.

*“Umm they did not say much, they just said they will give drugs and then they will do operation for her”* [28].

***Sub-theme 2: Disruption in communication***

Patients with cancer expressed that they had a limited meaningful interaction with their clinicians, leading to the provision of inadequate information and a poor understanding about their disease and treatment options due to disruptions in communication [28]. Moreover, other patients were in the consulting room, making it difficult to discuss sensitive information with patients.

*“Mmm, the people in the consulting room were many, they keep coming to interrupt and ask him [doctor] something and I was thinking me too I want to ask this question, but these people keep coming to the doctor to show him things, so the doctor did not get time, he could not get any time”* [28]*.*

***Sub-theme 3: Breaking Bad News***

Some patients indicated that although they are informed about their cancer diagnosis, how it is communicated makes them terrified [27,28]. They find it challenging to concentrate or receive additional information after receiving confirmation about their diagnosis.

*“I was expecting [the doctor] to tell me about my diagnosis in a sympathetic manner. However… she told me about my diagnosis in a manner that made me terrified… I even cried because it was a very difficult moment for me”* [27].

*“on that day, when the doctor told me about the results that it is cancer, hmm, all that I was thinking about was I am coming to die, therefore, all the things he said afterwards I did not hear anything at all. I heard him talking, but none made any meaning to me because all that kept coming back to my mind was the word cancer…”* [28].

***Sub-theme 4: Power Asymmetry- deference to Healthcare Providers***

Patients are unlikely to ask questions because they believe doing so would be perceived as undermining the authority of healthcare providers. They believe being silent and not asking questions depicts their respect for healthcare providers and enhances care delivery. They would rather expect healthcare providers to initiate discussions about their condition and treatment modalities.

*“…I know if anything at all it is doctors who are going to do everything, so I did not ask question”* [28].

*“I don’t do anything that will sound that I am challenging them and be asking too much questions, so I need to come down so they also take care of me”* [28].

***Theme 2: Communication and Information Needs and Expectations***

***Sub-theme 1: Information on Disease Condition***

Most of the studies in the scoping review emphasized that patients with cancer want information on the disease condition [15,25–27,29,31]**.** Patients often struggle to cope with cancer when they lack information on the condition they are suffering from. They expect healthcare providers to provide them with information about their cancer (diagnosis) so that they can understand the situation and help manage the symptoms they encounter, especially at home [15,25,29]. The information should also be provided in a language and simple terms they can understand.

*“I think the patient must know the kind of diagnosis she has. So, the name of the disease in the local language and the medical language should be given to the patient, and in as much [detail] as possible…”* [25].

It was also revealed that patients with cancer prefer to be informed about the stage and progress of the cancer [15,26]. Patients mostly do not know much about their conditions [29]. Patients anticipate that healthcare providers organizing educational programs about cancer may help them acquire adequate knowledge about such conditions.

*“For me, I expect the health providers to have an open discussion with me about the stage and progress of my disease. As I speak to you, I don’t fully understand my condition. I don’t know whether it is curable or not”* [15]

Moreover, patients expect to receive general information about cancer, including its meaning, causes and risk factors, and the cancer screening process [15,25–27,31]. This information helps to dispel misconceptions about cancer and enables patients to come to terms with the condition.

*“You know, cancer in our parts of the world, we have different views about what it really means. So, they need to know what it is about because there’s one question that most people normally ask. How did I acquire it? Is there something I can do? Or something I did wrong. So, there are a lot of questions that we need to help these people [women] to find answers to”* [25].

***Sub-theme 2: Information on Cancer Treatment***

The participants in the included studies of the scoping review expect to receive information regarding cancer treatment options and modalities and the cost involved [15,25,26,28,29]. Informing patients about the various treatment processes and modalities enables them to better cope with their condition [25]. Considering the cost of cancer treatment, patients need to be aware of those paid by the government through the National Health Insurance Scheme (NHIS) and those that need to be paid out of pocket to help them make informed decisions.

*“You need to let the patient know what is available in terms of treatment. No matter the stage…..something that can be done for the patient. So, I think in terms of information, that should be made available to the patient”* [25]

*“The doctors are not explaining things well for patients to understand. They need to inform us of the treatment modalities”* [29]**.**

***Sub-theme 3: Information on Prognosis***

A recurring pattern in the included studies was patients’ desire to gain a deeper understanding of their prognosis [15,25–27,29]. Patients need to receive information on the disease trajectory, including the likelihood of recovery, progression, or survival, as they become worried without such information. This information should be provided using simple, non-technical language while acknowledging emotions and responding with empathy*.*

*“And then apart from the diagnosis the patient deserves to know the prognosis”* [25].

*“Due to the lack of information, I have always been worried and thinking about what will happen next when I come for treatment”* [27]**.**

*“Sometimes, after the palliative chemotherapy, I experience some discomforts which I think are the side effects. So, it is my expectation that they will provide me with adequate information so that I know what to expect at the point of the treatment and care”* [15].

***Sub-theme 4: Ongoing Information***

The participants emphasized their expectation that palliative care service providers would maintain open communication with them throughout the progression of their condition or disease trajectory [15,25]. They desire ongoing dialogue that keeps them informed about their health status and treatment options and addresses their evolving concerns, questions, and emotional needs. Thus, maintaining open and transparent communication with them throughout the progression of the cancer.

*“As for the information they need it all the time. Each time they visit you still need to hammer on some of the points that maybe the patient has gone through”* [25].

***Sub-theme 5: Involvement in Decision-making***

Patients with cancer often felt excluded from the decision-making process about their care, according to the findings of the review. However, they expect to be actively engaged in every stage of the healthcare decision-making process [15,25,26]. They expect their preferences regarding treatment options, care plans, and other critical aspects of their health to be considered. This is essential to ensure that their care is aligned with their needs, values, and goals. This highlights the importance of patient-centered care, demonstrating respect for patients’ rights and dignity in cancer management.

*“I wish the doctors will involve me when making the decisions about the treatment. I do not know what informs the prescribed treatment. They do not talk to me about the treatment”*

*“I am not considered at all when it comes to decisions about my own treatment. I want to be part of this process”* [15]**.**

## Discussion

The review’s findings present the experiences and expectations of patients diagnosed with cancer and their information and communication needs during cancer treatment. To the authors’ knowledge, this is the first review to address patient communication preferences in cancer care in Ghana. Access to information is fundamental for making informed choices and decisions for patients. Effective communication involves prioritizing patients’ needs, ensuring a favorable information-sharing environment, and actively involving patients in their care [32]. Patients with cancer mainly depend on healthcare clinicians to understand the prognosis and treatment options to make informed decisions about their care [33].

The review illuminated that patients with cancer desire information on their disease conditions, prognosis, and treatment options. Patients with cancer yearn to know about the stage and progress of their cancer. They also expect general cancer information, including its meaning, causes, risk factors, management, and the cancer screening process. Similarly, studies conducted in other African countries like Nigeria [34] and Ethiopia [35] revealed that patients with cancer, specifically breast cancer, yearned for information about treatment options, benefits, and adverse effects of the various treatment types available. A study by Zafar et al. [34] assessing preferences regarding prognosis disclosure in a lower-middle-income country found that most adult patients with cancer preferred honest, empathetic disclosure of their disease prognosis and life expectancy, consistent with the findings of this review. This should be undertaken in a manner that meets their expressed information needs. Notwithstanding patients’ desire to understand their condition and treatment options as shown in the findings of this review, they hesitate to initiate such discussions, preferring healthcare professionals to broach these sensitive subjects as they believe they are the experts and do not want to undermine their authority. This may stem from the hierarchical structure of Ghanaian society, where individuals in positions of authority or seniority are viewed as wise and are traditionally consulted in decision-making processes [36]. Healthcare professionals are mostly considered as people of authority and experts by patients. This has been identified in other African countries like Nigeria [37] and in other Asian cultures like China where clinicians represent authority of medical knowledge and thereby patients valuing recommendations made by clinicians than their own preferences [38]. This demonstrates the need to empower patients through awareness and developing communication guidelines that are culturally sensitive.

Similar to the findings of this review, a systematic review assessing the information needs priorities among patients with cancer revealed that information concerning treatment alternatives was among the first three prioritized information needs of patients with cancer [39]. Another systematic review assessing the unmet needs of advanced patients with cancer also unraveled that the three most frequently reported needs of concern were psychological, physical, and healthcare service and information needs [40]. However, none of the studies included in these reviews were conducted in an African country.

It is important for healthcare professionals to discuss the prognosis, treatment options, and end-of-life preferences of patients with advanced cancers [41], as it leads to increased patient satisfaction and treatment outcomes [42]. The patients’ charter established by the Ghana Health Service asserts that patients are entitled to comprehensive information about their medical condition, including diagnosis, prognosis, treatment options, and potential complications [43]. However, despite the charter and patients’ desire for information on their disease conditions, prognosis, and treatment options, healthcare providers mostly do not provide such information. A study assessing the practices regarding communicating bad news in African oncology settings of which Ghana was included demonstrated that only a few clinicians have a consistent plan or strategy for delivering bad news to patients who have cancer [44]. It has also been identified in other countries like Tanzania that healthcare providers avoid or rarely provide such information due to clinicians’ discomfort, perceived patient reactions, and significant gaps in clinicians’ knowledge due to inadequate training in serious illness communication [45]. It is therefore important to train clinicians in communicating bad news and developing culturally appropriate frameworks or tools for delivering such news. Moreover, existing frameworks or programs, such as the serious illness conversation program, could also be adapted to the socio-cultural context of Africa to train clinicians and help them communicate bad news [46].

The review also revealed that patients with cancer expect ongoing information about their condition. Regularly assessing patients with cancer’s information needs is essential, as their priorities and preferences often evolve [35]. Ensuring patients receive sufficient information about their condition throughout the healthcare process can enhance trust between patients and clinicians, ultimately encouraging the effective utilization of healthcare services [47,48]. Moreover, facilitating continuous information sharing about cancer, available treatment modalities, anticipated outcomes or prognosis, and treatment costs helps in adherence to disease management instructions [41,49,50].

The scoping review further highlighted that patients with cancer prefer to be involved in decision-making. Similarly, Stacey and colleagues [51], in their study exploring patients with cancer about treatment decision-making, highlighted that only half of the patients in their study indicated that they were given options for their cancer treatment. Patients provided with choices were more actively involved in decision-making. However, a systematic review assessing the perceived and preferred level of patient involvement in decision-making revealed a wide variation in patients’ preferences, with a substantial number of patients perceiving a decisional role other than the preferred [52]. Additional efforts are needed to identify effective approaches to supporting patients with cancer’s increased involvement in decision-making regarding their care. Plewnia et al. [42] assert that healthcare providers ensuring shared decision-making, particularly regarding the treatment of patients with cancer, leads to enhanced patient satisfaction with care and improved treatment outcomes. Ethical, legal, and social imperatives strongly advocate for patients with cancer to be more active and engaged in their care decisions, fostering autonomy while collaborating with clinicians rather than remaining passive in the decision-making process [53].

### Strengths and limitations

The review had many strengths, including adopting a comprehensive search strategy, an independent screening process, and an appraisal by two authors. This is the first review conducted to explore the communication and information needs of patients with cancer in Ghana. Notwithstanding the strengths of the scoping review, only peer-reviewed studies were included. Peer-reviewed articles may not capture all relevant information, particularly from gray literature. Also, a limited number of studies have assessed the topic under review. Including studies with cancer at any stage could impact the findings, as communication practices or information needs may differ in cancer with curative intent and those in advanced stages that are mostly incurable.

### Recommendations

Addressing communication gaps and experiences is crucial for improving cancer care outcomes. Five key recommendations are made. 1. Effective measures should be implemented to identify relevant and culturally sensitive strategies and interventions that may enhance communication between healthcare providers and patients with cancer. 2. Patients with cancer should be provided with the necessary information about their condition. 3. Patients must be involved in decisions affecting their health. 4. Future studies should explore the current practices regarding communication between healthcare providers and patients with cancer. 5. Also, the barriers and facilitators to such communication should be assessed.

## Conclusions

This review has mapped the literature on the communication, information needs, and experiences of patients with cancer in Ghana. It has highlighted that patients with cancer have varied experiences and expect meaningful communication and information about their conditions. Shared decision-making has also been demonstrated as a key expectation of patients during their care. It is important to develop intervention-based strategies to enhance communication skills among healthcare providers and examine the impact of such interventions on patient satisfaction and quality of care. Addressing these gaps will provide a more comprehensive understanding and enhanced communication among clinicians and patients with cancer. Moreover, communication in cancer care in Ghana needs to be explored more, as only a few studies have been conducted in this important area of cancer management.

## Supporting information

S1 Appendix Preferred reporting items for systematic reviews and meta-analyses extension for scoping reviews (PRISMA-ScR) checklist. S1 Appendix presents the PRISMA-ScR checklist with corresponding pages where such information can be found. (PDF)

S2 Appendix Search strategy. S2 Appendix presents the search strategy used in the databases. (PDF)
