# Supplementary material for: Communication and information needs of patients with cancer in Ghana: A scoping review
Source: PLoS One. 2026 Feb 12;21(2):e0343094. doi: 10.1371/journal.pone.0343094 (PMC12900367; doi:10.1371/journal.pone.0343094)
Supplement: S2 Appendix — (PDF) [file pone.0343094.s002.pdf]

S2 Appendix. Search strategy

SN	Database	Query	Records Retrieved
1.	PubMed	Search: (((("neoplasms"[MeSH Terms] OR "neoplasm"[Title/Abstract] OR "cancer"[Title/Abstract] OR "cancers"[Title/Abstract] OR "malignancy"[Title/Abstract] OR "tumor"[Title/Abstract] OR "carcinoma"[Title/Abstract] OR "sarcoma"[Title/Abstract] OR "cancer treatment"[Title/Abstract]) AND "health communication"[MeSH Terms]) OR "communication"[MeSH Terms] OR "nonverbal communication"[MeSH Terms] OR "verbal communication"[Title/Abstract] OR "written communication"[Title/Abstract] OR "oral communication"[Title/Abstract] OR "information literacy"[MeSH Terms] OR "consumer health information"[MeSH Terms] OR "health information"[Title/Abstract] OR "information seeking"[Title/Abstract] OR "information"[Title/Abstract] OR "information needs"[Title/Abstract]) AND "Ghana"[Title/Abstract]) AND (2012/1/1:2025/1/1[pdat])	2151
2.	PsycInfo	1 cancer.mp. or exp *Neoplasms/ 88472<br>2 malignancy.mp. 1574	14

3 carcinoma.mp. 2725

4 tumo*.mp. 24061

5 sarcoma.mp. 581

6 cancer treatment.mp. 4704

7 oncology.mp. 14427

8 1 or 2 or 3 or 4 or 5 or 6 or 7 104927

9 exp *Health Information/ or information.mp. or exp *Information/ or exp *Information Literacy/ or exp *Information Seeking/ 579946

10 consumer health information.mp. 1074

11 information needs.mp. 2323

12 exp *Written Communication/ or exp *Oral Communication/ or exp *Verbal Communication/ or communication.mp. or exp *Communication/ or exp *Nonverbal Communication/ 520265

13 health communication.mp. 4640

14 9 or 10 or 11 or 12 or 13 995678

15 Ghana.mp. 4175

		16 8 and 14 and 15	16	
		17 limit 16 to yr="2012 - 2025"	14	
3.	CINAHL	XB ((“neoplasm*" OR "cancer*" OR "oncology" OR “cancer treatment” OR "malignancy" OR "tum#r" OR "carcinoma" OR "sarcoma")) AND XB (("health communication" OR "communication" OR "nonverbal communication" OR "verbal communication" OR "written communication" OR "oral communication" OR "information literacy" OR "consumer health information" OR "health information" OR "information seeking" OR "information" OR "information needs")) AND XB "Ghana"		40
4.	EMBASE	#49. #48 AND (2012:py OR 2013:py OR 2014:py OR 2015:py OR 2016:py OR 2017:py OR 2018:py OR 2019:py OR 2020:py OR 2021:py OR 2022:py OR 2023:py OR 2024:py OR 2025:py)	195	195
		#48. #33 AND #46 AND #47	211	
		#47. 'ghana':ab,ti	20,372	
		#46. #34 OR #35 OR #36 OR #37 OR #38 OR #39 OR #40 OR	2,647,515	

#41 OR #42 OR #43 OR #44 OR #45	
#45. 'information need':ab,ti	603
#44. 'information seeking':ab,ti	4,872
#43. 'medical information':ab,ti	18,323
#42. 'consumer health information':ab,ti	474
#41. 'information literacy':ab,ti	584
#40. 'information':ab,ti	2,284,550
#39. 'oral communication':ab,ti	892
#38. 'written communication':ab,ti	969
#37. 'verbal communication':ab,ti	4,321
#36. 'nonverbal communication':ab,ti	1,544
#35. communication:ab,ti	458,281
#34. 'medical information':ab,ti	18,323
#33. #25 OR #26 OR #27 OR #28 OR #29 OR #30 OR #31 OR #32	7,152,508
#32. 'sarcoma':ab,ti	134,881

#31. 'carcinoma':ab,ti	1,088,768
#30. tumor:ab,ti	2,232,516
#29. 'malignancy':ab,ti	328,397
#28. 'cancer therapy':ab,ti	93,977
#27. 'oncology':ab,ti	285,668
#26. cancer:ab,ti	3,522,498
#25. 'neoplasm'/exp OR 'neoplasm':ab	6,904,560
#24. #9 AND #22 AND #23	211
#23. 'ghana':ab,ti	20,372
#22. #10 OR #11 OR #12 OR #13 OR #14 OR #15 OR #16 OR #17 OR #18 OR #19 OR #20 OR #21	2,647,515
#21. 'information need':ab,ti	603
#20. 'information seeking':ab,ti	4,872
#19. 'medical information':ab,ti	18,323
#18. 'consumer health information':ab,ti	474
#17. 'information literacy':ab,ti	584

#16. 'information':ab,ti	2,284,550
#15. 'oral communication':ab,ti	892
#14. 'written communication':ab,ti	969
#13. 'verbal communication':ab,ti	4,321
#12. 'nonverbal communication':ab,ti	1,544
#11. communication:ab,ti	458,281
#10. 'medical information':ab,ti	18,323
#9. #1 OR #2 OR #3 OR #4 OR #5 OR #6 OR #7 OR #8	7,152,508
#8. 'sarcoma':ab,ti	134,881
#7. 'carcinoma':ab,ti	1,088,768
#6. tumor:ab,ti	2,232,516
#5. 'malignancy':ab,ti	328,397
#4. 'cancer therapy':ab,ti	93,977
#3. 'oncology':ab,ti	285,668
#2. cancer:ab,ti	3,522,498
#1. 'neoplasm'/exp OR 'neoplasm':ab.ti	6,904,560

5.	Web of Science	("neoplasm*" OR "cancer*" OR "oncology" OR “cancer treatment” OR "malignancy" OR "tum#r" OR "carcinoma" OR "sarcoma") (Topic) and ("health communication" OR "communication" OR "nonverbal communication" OR "verbal communication" OR "written communication" OR "oral communication" OR "information literacy" OR "consumer health information" OR "health information" OR "information seeking" OR "information" OR "information need*") (Topic) and "Ghana" (Topic)	138

S2 Appendix. Search strategy. S2 Appendix presents the search strategy used in the databases.
